# Droughts and conflicts during the late Roman period

**DOI:** 10.1007/s10584-025-03925-4

**Published:** 2025-04-16

**Authors:** Charles Norman, Lothar Schwinden, Paul Krusic, Andreas Rzepecki, Tatiana Bebchuk, Ulf Büntgen

**Affiliations:** 1 Department of Geography, University of Cambridge, Cambridge, CB2 3EN UK; 2 Generaldirektion Kulturelles Erbe Rheinland-Pfalz, Direktion Rheinisches Landesmuseum Trier, 54290 Trier, Germany; 3 Global Change Research Institute, Czech Academy of Sciences, Brno, 603 00 Czech Republic; 4 Department of Geography, Faculty of Science, Masaryk University, Brno, 611 37 Czech Republic

**Keywords:** Barbarian Conspiracy, Climate change, Conflict, Drought, Interdisciplinary, Roman Empire, Resilience

## Abstract

Despite continuous investigation, reasons for both the abandonment of Roman Britain around 410 CE, and the separate collapse of the Western Roman Empire in 476 CE remain unclear. Here, we use tree ring-based climate reconstructions and written documentary sources to show that a sequence of severe summer droughts from 364 to 366 CE not only contributed to prolonged harvest failures and food shortages, but also played a role in the ‘Barbarian Conspiracy’, a catastrophic military defeat for Roman Britain in 367 CE. In line with contemporary reports from the historian Ammianus Marcellinus, this pivotal event in pre-modern history coincided with anomalous coin hoarding, and a gradual depopulation of Roman villas and towns. Expanding our climate-conflict analysis from Roman Britain as a case study to the entire Roman Empire and the period 350–476 CE reveals clear linkages between years in which battles occurred and preceding warm and dry summers. Based on these findings, we develop a mechanistic model to explain the vulnerability of agrarian societies to climate variability, whereby prolonged droughts cause harvest failures and food shortages (dependant on societal resilience) that lead to systematic pressure, societal instability, and eventually outright conflict.

**Supplementary Information:**

The online version contains supplementary material available at 10.1007/s10584-025-03925-4.

## Introduction

The role of climate on conflict initiation is becoming increasingly evident through growing scholarship, and both temperature and precipitation anomalies have been shown to increase the risk of conflict at different spatiotemporal scales (Burke et al. 2015; Lee and Qiang 2023; Ljungqvist et al. 2020; Mach et al. 2019, 2020). This poses challenges for contemporary societies under anthropogenic climate change, as water and food resources are likely to become scarcer and the risk of national and international conflict increases (Bowles et al. 2015). Robust mechanisms linking climate and historical conflict are, however, difficult to construct, and high-resolution proxy evidence is needed to build holistic understanding of the putative climate-conflict nexus (Buhaug 2015; Koubi 2019; Mach et al. 2020). In particular, establishing linkages in pre-modern periods is limited by the availability of historical, archaeological and paleoclimatological data. Nonetheless, exploring possible interactions between climate and conflict may deepen our understanding of direct and indirect linkages between past societies and their environments (Jun and Sethi 2021; Kennett et al., 2022; Kushnir and Stein 2019). Increasing numbers of annually resolved early Common Era (1–500 CE) climate proxy archives are now allowing climate-conflict interactions to be investigated, while improved archaeological datasets from the Western Roman Empire provide an ideal case study in which to assess the role of climate extremes on societal perturbations (Jongman et al. 2019).

**Fig. 1:**
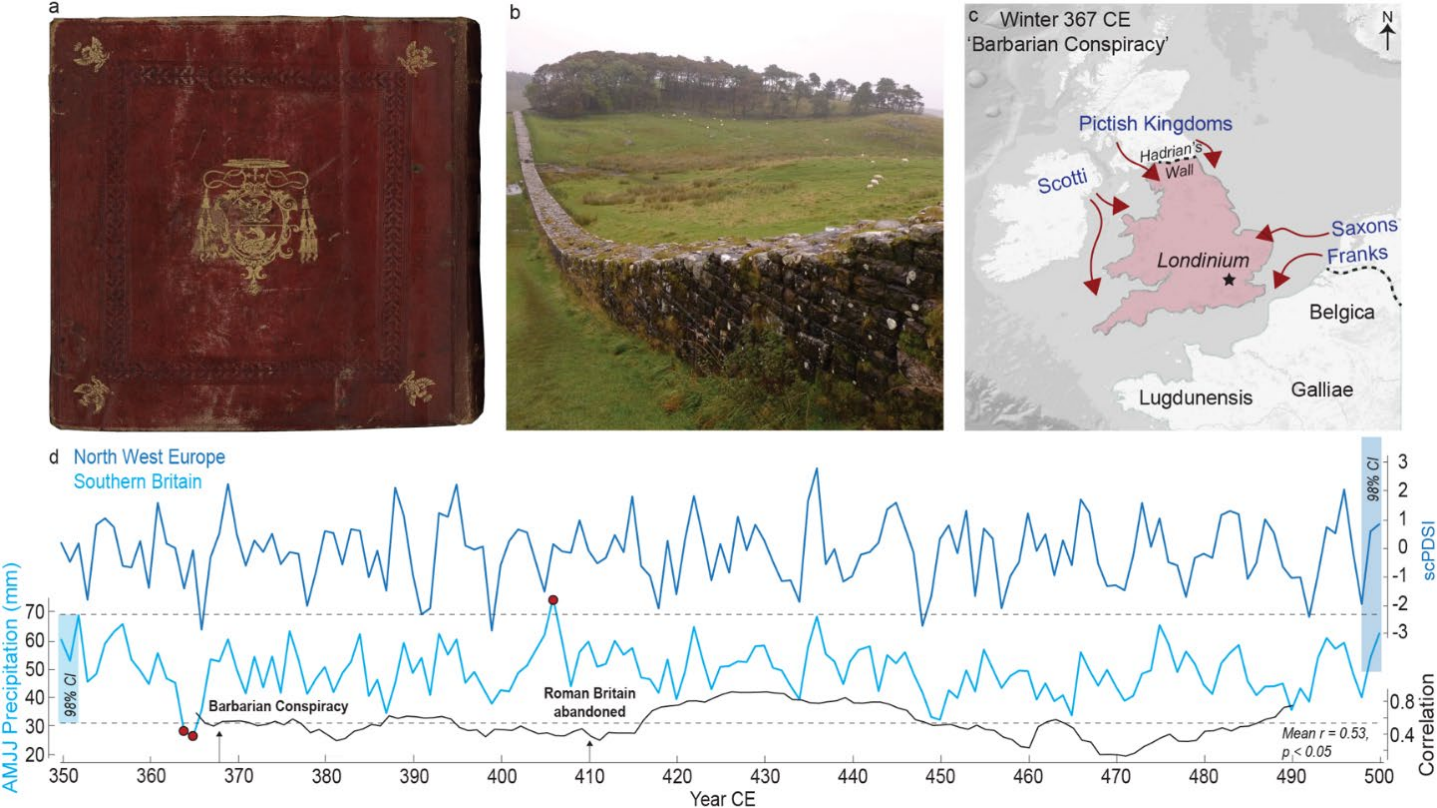
Climatological and historical background for the Barbarian Conspiracy. (**a**) *Vaticanus Latinus* 1873, one of two surviving independent manuscripts of Ammianus’ *Res Gestae* in which the events of the 367 CE Barbarian Conspiracy are described, re-discovered in the Abbey of Fulda, Germany in 1417 CE (**b**) Hadrian’s Wall, one of the sites where major incursions likely occurred after the garrison rebelled and invaders were allowed to pass through. (**c**) The broad movements of tribes outlined by Ammianus in the winter of 367 CE. (**d**) Early Common Era hydroclimatic records covering 350–500 CE over varying spatial scales. Dark blue shows an OWDA (Cook et al. 2015) extract from northwest Europe (47–54 N, − 1E–11E), while light blue is a precipitation reconstruction using OWDA-derived *Quercus* data specifically from Southern Britain. Three statistically significant extreme events (red dots) identified from 98% confidence intervals, two falling in 364 and 365 (*p* < 0.01), with 366 CE just above the threshold for the entire period (350–500 CE). The 98% CI for the NW European scPDSI record is not exceeded in the period shown

The ‘Barbarian Conspiracy’ in 367 CE was one of the most severe threats to the control of the Roman Empire over its most northerly province since the Boudiccan revolt three centuries earlier (De la Bédoyère 2014). The event was documented by the contemporary soldier, historian, and writer Ammianus Marcellinus in the *Res Gestae* (Ammianus XXVII. 8, Fig. 1a). Though his interpretations of past and present proceedings appear somewhat capricious (Weisweiler 2014), his descriptions of historical events, including the ‘Conspiracy’, are now widely regarded as reliable (Blockley 1996; Kelly 2008). Literary and archaeological information provide the following general chronology of events: In the winter of 367 CE parts of the garrison on Hadrian’s wall rebelled and allowed the Picts, a heterogenous group of people from northern Britain, to attack the Roman province by land and sea (Frend 1992; Fig. 1b). Simultaneously, the Scotti from modern-day Ireland invaded the west of Britain, and Saxons from the continent also landed in southern Britain, defined henceforth as the area south of the Wash/Severn line (Fig. 1c). The commander of the ‘Saxon Shore’, *Comes* Nectaridus, was killed and a senior general named Fullofaudes, possibly the *Dux Britanniarum* or commander of northern forces, was captured. With the army essentially beheaded, some soldiers were reported to have deserted and joined the invaders (Blockley 1980). Throughout the spring and summer of 367 CE, small groups roamed and plundered the Romano-British countryside (Southern 2004). The breakdown of Britain into anarchy represented a disastrous situation for the Roman administration on the continent (Álvarez-Jiménez 2013).

Valentian I, Emperor of the Western Roman Empire from 364 to 375 CE, sent the senior generals Severus and Jovinus to restore order in Britain, though this strategy failed (Bartholemew 1984). The “pressing necessities of the situation” led to another highly respected military officer, *magister militum* Theodosius the Elder, being called to Britain (Ammianus, XXVII. 8). Under Flavius’ leadership and accompanied by his son the later Emperor Theodosius I, Roman authority was restored after two years (Blockley 1980). The extent of the destruction and subsequent restoration is a matter of continual debate, and some historians argue that the province never fully recovered (Faulkner and Reece 2002; Morris 2021; Todd 2003). Little evidence exists for systematic urban destruction, though the abandonment of Roman villas and towns across southern Britain has been associated with the downturn after the ‘Conspiracy’. This is partially unsurprising as raiders, unlike settlers, leave little archaeological evidence, and archaeologists tend to interpret signs of fire destruction as accidental rather than deliberate (Morris 2021). High rates of coin hoarding in the 360s was likely a response to persistent external threats nevertheless, while a sudden decline after 368 CE reflects the restoration of Roman military control (Black 1987; Rance 2012; Álvarez-Jiménez 2013; Bland 2018) (Fig. S1). The withdrawal of Rome from peripheral Britain one generation later is perhaps suggestive of the scale of destabilisation that the island experienced (Clearly 2000; Ward-Perkins 2005).

Similarly, the extent to which the ‘Conspiracy’ was orchestrated is uncertain, though it’s possible that the exiled rebel Valentinus, brother-in-law to Maximus, close friend of Emperor Valentinian I, played some unifying role for the tribes.

The ‘Barbarian Conspiracy’ cannot be understood as a stand-alone event and must be placed in the wider context of an unstable period for the entire Roman Empire (Crawford 2016). Though nothing to match the crises of the 5th century CE, much of Europe in the mid- 4 th century was experiencing extensive geo-political, socio-economic, and cultural challenges, with roots in the crises of the 3rd century and even earlier (Salzman 2021; Schmidt-Hofner 2017). As Roman Britain was already suffering from external attacks, the province was particularly susceptible to the wider turmoil across the Empire (Fleming 2021; Salway 2015; Wacher 2017). Chronicles suggests that the 360s were chaotic in Britain, with numerous small incursions across Hadrian’s wall from the Picts and across the Irish Sea from the Scots (Frend 1992). By 365 CE, the Saxons and ‘Attacotti’ were building further pressure from the continent and potentially Ireland. These encroachments, driven in part by the waning control of the Western Roman Empire, were worsened by decades of soldiers being withdrawn from Britain to fight in other conflicts across the Empire (Southern 2004). Contemporary chroniclers additionally recognised the growing financial difficulties of the provincials in Roman Britain (*De rebus bellicis* in Thompson 1952). These factors alone, however, fail to explain the abrupt and seemingly coordinated nature of the ‘Barbarian Conspiracy’ (Pace 2021). Interdisciplinary investigations are therefore needed to deepen our understanding of the possible interplay between a range of natural and societal processes that may, or may not, have contributed, directly or indirectly, to such regional collapses up to the general demise of the Western Roman Empire (Izdebski et al. 2016; Harper 2016; Zonneveld et al. 2024).

Here, we compile annually resolved and absolutely dated tree-ring chronologies from western Europe to reconstruct regional temperature and hydroclimate variability before, during and after the ‘Barbarian Conspiracy’ in 367 CE. Moreover, a subset of tree-ring width chronologies initially used in the Old World Drought Atlas (OWDA; Cook et al. 2015), is re-combined to reconstruct April–July precipitation totals over southern Britain during the period of interest. These records are augmented alongside historical documentary sources, and recently compiled data on coin hoards to gain insights into societal cohesion, herein defined as the absence or presence of conflict, and strength of public institutions and power hierarchies within a society. We then use a newly compiled record of Roman conflict to expand our climate-conflict analysis from Britain across the entire Roman Empire. In so doing, we explore if internal and external battles between 350 and 476 CE were preceded by unusual climate conditions. Our newly obtained empirical evidence allowed us to develop a mechanistic model to explain if climate has contributed to food shortages and subsequent conflict in the late Roman world.

## Data and methods

### Historical and climate data

The primary source of information for the ‘Barbarian Conspiracy’ is book XXVII (27) of *Res Gestae* by Ammianus Marcellinus (see Blockley 1996). While these descriptions by Ammianus are short and lack important chronological, military, or geographical details, sufficient information has been extracted by historians so a causative outline of the ‘Barbarian Conspiracy’ can be constructed. Additional semi-contemporary information comes from works such as ‘*Notitia Dignitatum’*, a late Roman document that provides information on military organisation in Roman Britain (White 2009). Archaeological evidence for the ‘Barbarian Conspiracy’ is limited, though information can be gained from a series of fortification constructions built in the recovery period of 368/369 CE (Burnham 2014). The nature of exploring an event almost 1700 years ago means that uncertainties remain concerning the timing, scale, order and response to the ‘Barbarian Conspiracy’, though combining various recent and contemporary sources of information has allowed a narrative of events to be constructed with sufficient temporal resolution to undertake climate-conflict analysis (Blockley 1980).

Our new hydroclimatic reconstruction for the ‘Barbarian Conspiracy’ was based on a subset of living and relict oak (*Quercus* spp.) tree-ring width (TRW) series that we collated from the OWDA (Cook et al. 2015). This subset contains data from southern Britain and northern France from 48–52°N and 3°W to 2°E. The composite TRW dataset, at its maximum incorporating 2831 individual tree-ring series, spans the period 288–2009 CE and was detrended with length adaptive cubic smoothing splines at 2/3 frequency cut-off at the individual series length, in order to remove age-related trends. Artificial variance changes in the final TRW chronology were stabilised with a 31-year moving window approach during which the standard deviation was subtracted from the index value (Büntgen et al. 2024). The final TRW chronology was then correlated against a wide range of climate parameters using the KNMI Climate Explorer (Sitko et al. 2016).

In agreement with previous studies (Briffa and Wigley 1985; Cooper et al. 2013), the greatest correlation (*r* = 0.50–0.60; *p* < 0.01) was found with spring-summer precipitation (CRU TS 4.06, 1901–2009 CE), the season most relevant for oak growth in southern Britain (Wilson et al. 2013). The four-month season April-July (AMJJ) was established as producing the highest correlation with the precipitation data. Climate stations within southern Britain and northern France were evaluated for correlation between their modern instrumental AMJJ precipitation data and our AMJJ chronology over the period 1965–2000 CE. The extent of correlation ranged from Exeter (*r* = 0.32, *p* < 0.01) to Yeovilton (*r* = 0.62, *p* < 0.01). Once Yeovilton had been identified as the site with the highest correlation, calibration and verification was carried out over the split periods 1965–1982 and 1983–2000. This produced *r* values of 0.71 for 1965–1983, 0.62 for 1983–2000, and an overall *r* value of 0.62 (*p* < 0.01) for the entire calibration period of 1965–2000 (compared to spearman’s value of 0.53; *p* < 0.01).

The robustness of the model was tested using a series of measures in line with standard dendroclimatological methodologies described by Cook et al. (2015). Residuals were calculated and plotted to ensure that longer term trends had been removed, while kurtosis and skewness values of -0.67 and 0.02, respectively, demonstrate a normal univariate data distribution. Once tested for its ability to reproduce the instrumental data, our model was applied to the TRW chronology values from 288 to 500 CE, creating an AMJJ precipitation reconstruction for southern Britain during the late Roman period (Fig. 1d).

### Network analysis and statistical verification

Further to our climate-conflict assessment of the 360s in Roman Britain, we extended our study across the entire Roman Empire from the reign of Valentinian I (365 CE) to the deposition of Romulus Augustulus (476 CE). We therefore compiled a network of 106 battles from across the Roman Empire, all of which are major engagements with regional implications (Schwinden 2022). Each battle includes a description, location and annual or sub-annual dating for categorisation, as well as the relevant historical sousrces (Schwinden 2022). Environmental data used is an extant set of annually resolved and absolutely dated dendrochronology-based climate reconstructions for Europe (Table 1). To explore how these records interacted with the conflict network, a novel yet simple methodology was utilised: ‘event-based aggregation analysis’, which summarizes data based on a particular event. Firstly, the battle dataset was divided between ‘East’ and ‘West’ based on the 395 CE division of the Roman Empire in order to ascertain different environmental relationships across the broad continental gradient (Mitchell and Greatrex 2023). For each individual battle, the spatially relevant climate records were identified from our set of possible reconstructions (Table 1). Climate values for ten years before and four years after battle were extracted, then normalised against themselves to obtain a mean of 0 and standard deviation of 1 (Z-scores). For each region the 15-year-long standardised climate records were aggregated in a variation of Superposed Epoch Analysis (SEA) (Rao et al. 2019). At this stage a standard deviation threshold was identified, based on data distribution, and all those that did not reach the ‘extreme’ threshold were excluded. The resultant is a sum of climate ‘extremes’ prior to and after battles in the Western and Eastern Roman Empire.

**Table 1:** Tree ring-based climate records used in this study. Asterisks indicate which are used in the analysis of the entire Empire

Type	Region	Period	Journal	Source
Summer Temperature and precipitation	Central Europe	500 BCE–2000 CE	*Science*	Büntgen et al. 2011*
Summer Temperature	Eurasia	1–2010 CE	*Dendrochronologia*	Büntgen et al. 2020*
Summer Hydroclimate	Central Europe	75 BCE–2018 CE	*Nature Geoscience*	Büntgen et al., 2021
scPDSI reconstruction	Eurasia	1–2012 CE	*Science Advances*	Cook et al. 2015*
Summer Temperature	North Scandinavia	16 BCE–2006 CE	*Journal of Quaternary Science*	Esper et al. 2017*
Summer Precipitation	Southern Britain	350–500 CE	This study	

One-sided Z-score tests were used to determine the significance of our observed extreme climate frequencies compared to an independent baseline value (-10 to 4 excluding -4 to 0). This baseline period was chosen because of its independence from our ‘signal period’, the adequate size providing a stable and robust baseline, and we expect these years to be representative of ‘normal’ conditions. Our results allow high confidence that the observed differences in climate extreme frequency between the ‘baseline’ period and years within the signal period are statistically significant. In particular, the year -3 has the greatest frequency of significant climate years, suggesting it is a critical precursor to conflict initiation, while the year ‘0’ is often less significant, demonstrating that a lag period is a common feature of climate-conflict interactions.

## Results

Both the OWDA-based drought reconstruction for northwestern Europe and our new precipitation reconstruction for southern Britain suggest that the hydroclimate preceding the ‘Barbarian Conspiracy’ in 367 CE was exceptional (Fig. 1d). Southern Britain experienced an unusual sequence of three remarkably dry summers from 364 to 366 CE, with 364 CE (*p* = 0.011) and 365 CE (*p* = 0.006) exhibiting precipitation levels well below the 98% confidence interval of the long-term average (Fig. 1d). Across the entire period 350–500 CE, the reconstructed average April-July monthly precipitation total is 51 mm, while 364, 365 and 366 CE only experienced 29 mm, 28 mm and 37 mm, respectively. In other words, these three years exhibited 58%, 54% and 72% of the average amount of spring-summer precipitation across the main growing season in southern Britain. When considering northwestern Europe as a whole (47–54°N and 1°W to 11°E), the dry summers are less marked. The summer of 366 CE is the driest with a self-calibrated Palmer Drought Severity Index (scPDSI; Van der Schrier et al. 2013) of -2.2 (moderate drought), though 365 CE and 367 CE have scPDSI values of -0.4 and -0.6, respectively (Fig. 1d).

**Fig. 2:**
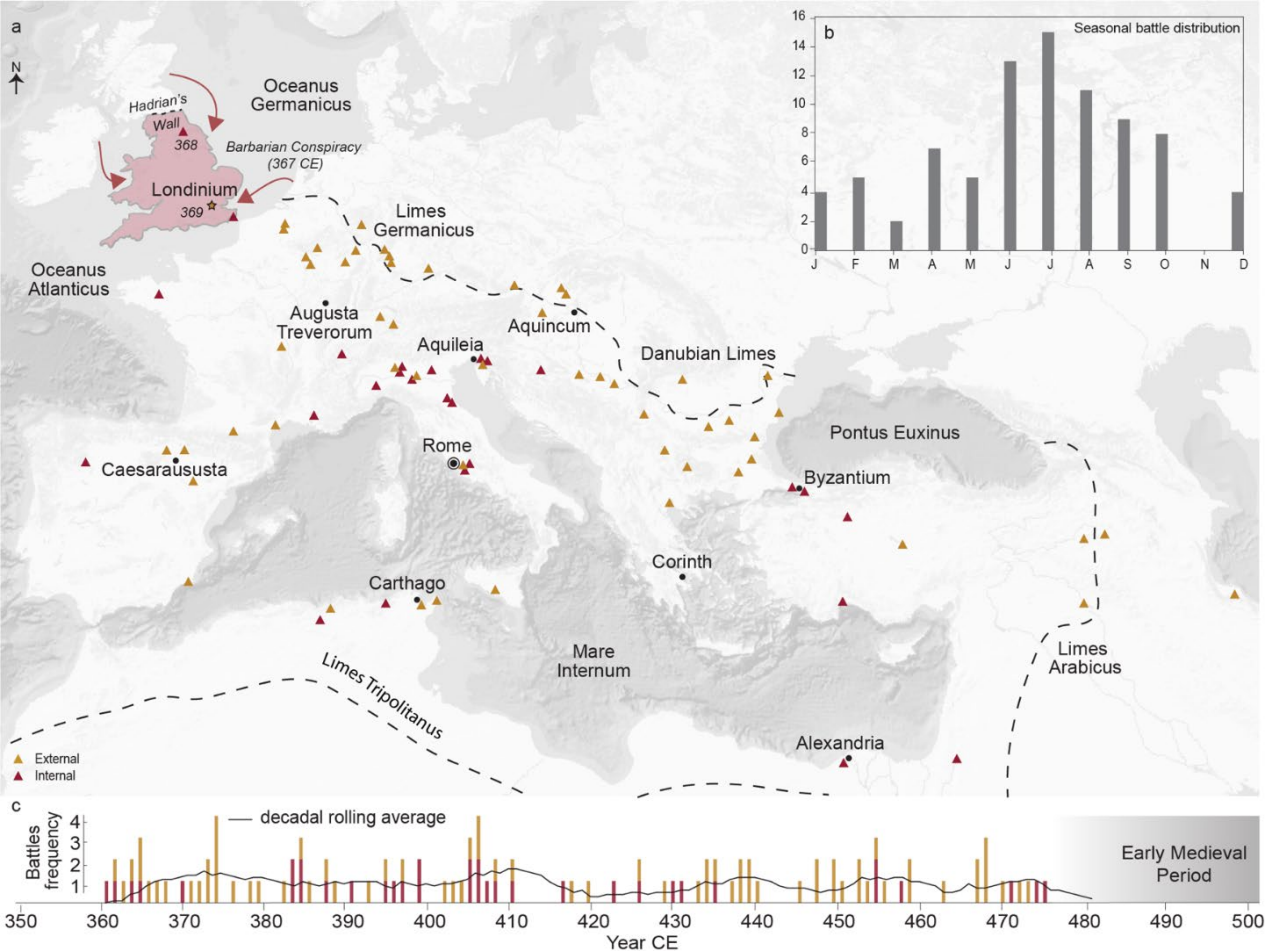
Spatial and temporal distribution of 106 battles in the late Roman period from 360 CE to 476 CE from Schwinden (2022). (**a**) Conflict divided into internal (civil conflicts between Roman armies; red) and external (conflicts with people outside of the Empire; yellow). Province of Roman Britain (41–410 CE) highlighted in red, with two battles fought in response to the 367 CE Barbarian Conspiracy shown. The ‘Barbarian Conspiracy’ of 367 CE is excluded for being a multi-year period of incursions and pillaging, rather than an individual battle. Intra-annual battle distribution of all sub-annually dated battles (**b**) and temporal distribution of battles from 360 to 476 CE, with decadal rolling averages (**c**)

The following section moves on from the ‘Barbarian Conspiracy’ to the whole Roman Empire and the period 365–476 CE (Fig. 2). Our high-resolution conflict data cover the entire climatological gradient of the Roman Empire from York, where a battle occurred in 368 CE between *magister militum* Theodosius and the usurper Valentinus, to Sefid-Rud on the Caspian Sea. The majority of late Roman battles took place on the European continent, particularly around the Rhine and Danube frontiers as well as the Italian peninsula. Battles in the provinces of North Africa and the Near East are relatively sparce during the late Roman period. Distinction patterns exist when comparing internal battles (civil wars) and external battles – those that occurred between Roman forces and forces outside of the Empire (Fig. 2). Internal conflicts were typically concentrated around the Italian peninsula, the centre of power in the Empire, and cities such as Rome, Aquileia and Byzantium saw particularly high instances of conflict. Battles on the frontiers of the Roman Empire were predominantly external and fought against tribes such as the Goths and Vandals. Our data confirms that seasonality was a key feature of late Roman conflict - the majority of battles fell within the drier months of summer, with significantly fewer in the winter months (Fig. 2b). A Chi-Squared test for uniformity confirms a statistically significant, seasonal aspect to Roman conflict (*p* < 0.001). The temporal distribution of these battles (Fig. 2c) from 360 CE until the end of the Empire in 476 CE is relatively uniform, with battles taking place in most years. There are, however, periods in which battles cluster such as the 400s CE, that contrast to relatively peaceful periods such as the 410s CE.

**Fig. 3:**
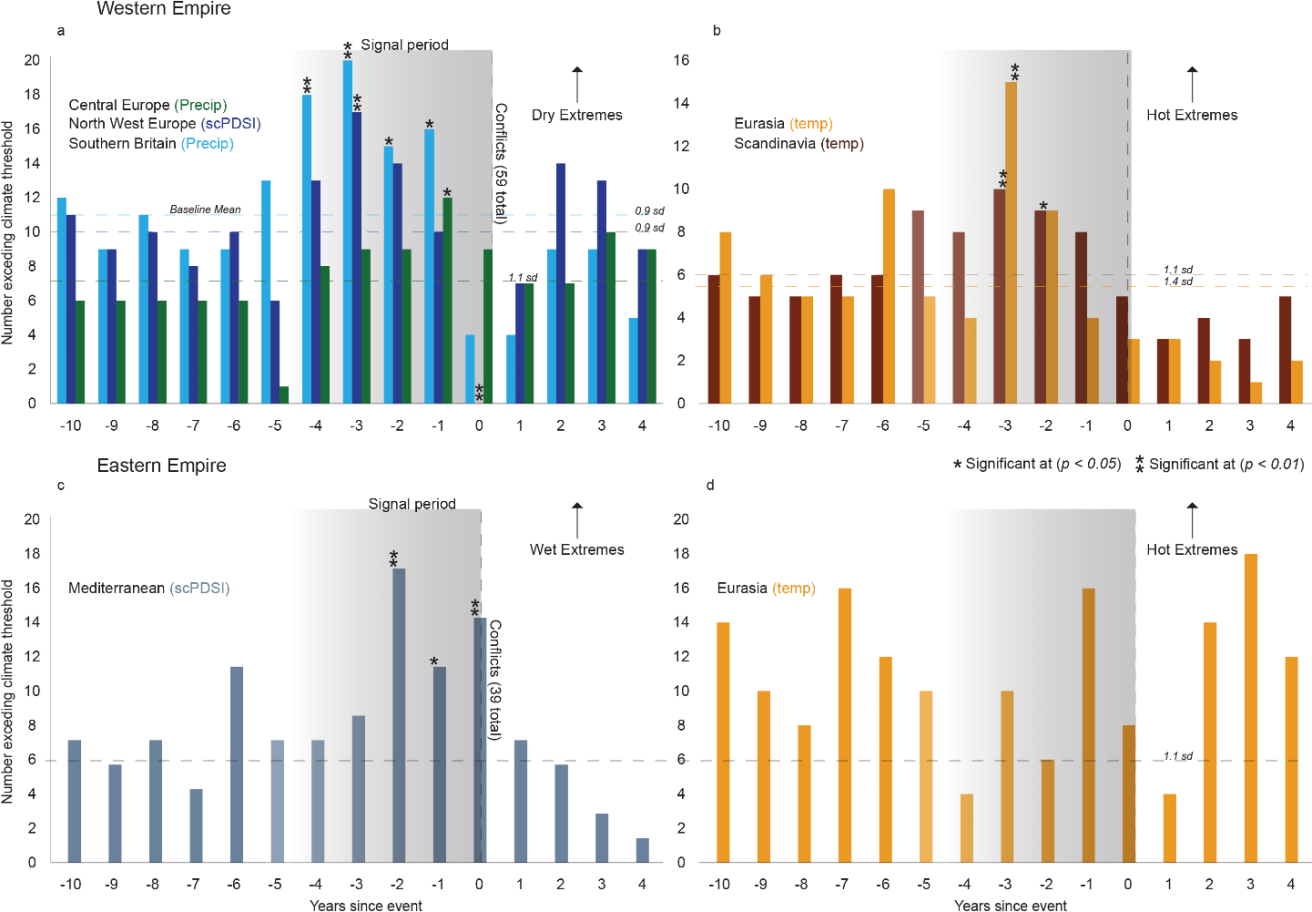
Climate extremes prior to conflict events for the Western and Easter Roman Empire. (**a**) Plot of years before or after 59 conflicts in the Western Empire exceeding a dry threshold of 0.9/1.1 standard deviations away from the mean, demonstrated by two independent climate records. (**b**) Plot of years exceeding a hot threshold of 1.1/1.4 SD from the mean demonstrated by two independent climate records. (**c**) Plot of years before and after 39 conflicts in the Eastern Empire exceeding a wet threshold of 1.1 SD from the mean. (**d**) Plot of years exceeding a hot threshold of 1.1 SD from the mean in the Eastern Empire. Dotted lines indicate the average number of conflicts exceeding the climatic thresholds across the 59 western and 39 eastern battles respectively. Significant deviations from the baseline (*p* < 0.05) are marked with an asterisk (*), and those at *p* < 0.01 are marked with two asterisks (**). Baseline values represent years -10 to -5 and 1 to 4

A relationship between years in which these battles took place and the climate that preceded them is evident in the climate-conflict aggregate analysis (Fig. 3). For 59 battles that occurred in the Western Empire, there is a significant increase in years exceeding the ‘dry’ climate threshold (0.9/1.SD) prior to the ‘conflict year’ (Fig. 3a). Our precipitation reconstruction, the OWDA excerpt from northwestern Europe, and an independent central European climate reconstruction jointly show this increasing prevalence of years exceeding the climate threshold between one and five years prior to the battle. The greatest frequency of dry summers is found three years prior to the battles (*p* < 0.01), though the central European record shows it as one year prior (*p* < 0.05). The ‘battle year’ itself has very few which surpass the dryness threshold in any of the climate records, and the scPDSI record shows a statistically significant omission of dry years coinciding with battles (*p* < 0.01). Similarly, occurrences of ‘exceedingly’ hot years increased in the years prior to conflict as shown by the two independent temperature records (Fig. 3b). For instance, three years prior to the conflict year the records exhibit ten and 15 exceedingly hot years respectively compared to a wider average of six (*p* < 0.01). Though smaller than the change associated with hydroclimatic conditions prior to these battles, a link between warm summers and conflict is clear. These results demonstrate that battles in the late Western Empire were, more commonly than not, preceded by warm and dry conditions, while the conflict years typically exhibited cooler, wetter conditions.

From the network of 39 battles across the eastern part of the Roman Empire, the occurrence of years exceeding the wet threshold increases prior to conflict (Fig. 3c). The average number of years exceeding the wet threshold (1.1 SD) is six, while two years prior to the battle year has 17 exceeding the threshold (*p* < 0.01). Unlike in the Western Empire where the battle year saw more typical seasonal conditions, in the East the battle year itself also has numerous years exceeding the wet threshold for the summer. Again, contrasting the Western Empire, the relationship between temperature and battles in the east is non-existent, where no statistically significant relationship can be identified in the number of warm years prior to the aggregate battle year (Fig. 3d).

## Discussion

### Placing the ‘Barbarian Conspiracy’ in an environmental context

Comparisons between our paleo-results and modern precipitation totals allow for a greater contextualisation of the identified dry years. Mean monthly AMJJ precipitation of 51 mm aligns closely with modern station observations from southern Britain, which average 53 mm over 1836–2024 CE (Met Office; Alexander and Jones 2000) (Fig. 1d). During this period, droughts of a similar magnitude (< 30 mm AMJJ) occurred only seven times, or one for every 27 years, and heavily skewed towards recent decades. Many of these years (1844, 1870, 1921, 1976, 1995, 2010, 2022 CE) had significant agricultural and societal impacts, such as in 1844 CE when almost no spring rain fell at all, causing widespread and persistent crop failures and grain and grass shortages across southern Britain as reported by 184 newspaper entrees (UKCEH, Historic Drought Explorer 2024). Similarly severe effects on agricultural production occurred in 1921 (Van der Schrier et al. 2021), 1976 (Carter 1978), 2010 (Kendon et al. 2013) and 2022 CE (Barker et al. 2024). These modern droughts demonstrate the role and severity of such events in southern Britain, and subsequent vulnerability of agricultural productivity. In contrast to the recent period, this magnitude of precipitation occurs only twice in our reconstruction (350–500 CE), in 364 and 365 CE, or at a frequency of one in every 75.5 years. Exceptionally, however, they occur in consecutive years which is unseen in the recent period (1836–2024 CE) and therefore implies a much stronger effect on the productivity and functioning of both natural and agricultural systems, with subsequent consequences for society.

It is important to gain an understanding of agricultural practises in the later Roman period in Britain, where the Empire’s occupation led to a large increase in the area of land under cultivation that was crucial for feeding and maintaining order in the army, particularly on Hadrian’s Wall (Allen et al. 2017; Southern 2004). For the first two centuries grain was imported, though by the third century Britain, predominantly the south-east, was a net exporter to regions such as the Rhine frontier (Potter and Johns 1992). The main crops were spelt wheat (*Triticum spelta L.*) and six-row barley (*Hordeum vulgare L.*) (Allen et al. 2017). Unlike the presently favourable ‘winter sown’ cereals, many of these crop varieties were sown in the spring to reduce weed colonisation (Allen et al. 2017; Lepetz and Matterne, 2018; Murphy et al. 2000). Tillage, prior to mechanisation, was also challenging in wet winter soils, making spring-sown a more viable option for Britain as a wet province (Murphy et al. 2000). This comes at a risk; spring sown crops are particularly vulnerable to late spring and early summer moisture deficits, and early summer droughts can lead to total crop failure (Pribyl 2020; Taheri 2011). Since various climate proxy archives suggest that the Roman period in Britain was relatively warm and dry (Charman 2010), droughts were likely to have been felt more severely (Dark and Dark 1998).

The reconstructed spring-summer precipitation deficits from 364 to 366 CE thus would have impacted spring-sown crop growth substantially, triggering poor harvests in one or several years between 364 and 366 CE. It also seems that the drought-like conditions were most severe in southern Britain, with other parts of northwest Europe exhibiting more normal hydroclimatic conditions (Fig. 1d). Poor harvests would have reduced the grain supply to Hadrian’s Wall, providing one plausible motive for the rebellion of the ‘Areani’, cited by Ammianus as playing a central role in allowing the Picts into northern Britain (Ammianus XXVIII. 8. in Hind 1983). This suggestion of drought-driven grain deficits can be corroborated by extracts from chroniclers. Zosimus, in *Historia Nova III*, writes that in 359 CE Britain was preparing six hundred ships to transport grain to the lower Rhine, though a contemporary, the anonymous author of *De Rebus Bellicis*, recognises the growing misery of the provincial population (*Zosimus*,* Hist. Nova* in Frend 1992). Crucially, by 367 CE, Ammianus describes the population of Britain as in a state of extreme need, or the “utmost conditions of famine” (Ammianus XXVII. 8 in Frend 1992). The likely grain deficits also provide a reasonable contributary factor for other desertions cited during this period, given the role of grain in the contract between soldiers and the army, and thus a general weakening of the Roman army in Britain at the time (Ammianus XXVII. 8; Erdkamp 2005). Hence our new hydroclimate reconstruction, together with archaeological, agricultural and literary knowledge from Roman Britain suggest that these exceptionally dry conditions from 364 to 366 CE contributed to the 'Barbarian Conspiracy', with agriculture as a key nexus (Parker 2013).

### Rethinking the causal mechanism of the ‘Barbarian Conspiracy’

There now seem to be multiple possible mechanisms for the ‘Conspiracy’ itself, one of which is that for peripheral tribes, the military and societal breakdown of cohesion in Roman Britain provided an opportunity to invade the province *en masse* with the intention of raiding rather than conquest – a ‘pull’ theory. Alternatively, the conflict could have arisen, or at least reinforced, from famines in regions external to Roman Britain, forcing them to enter the province to meet their needs – a ‘push’ theory. The ‘pull’ mechanism is favoured for several reasons. Firstly, the hydroclimatic drought was likely restricted to southern Britain, with the OWDA-based record suggesting more normal conditions for continental tribes surrounding the province (Fig. 1d). Secondly, extracts from chroniclers suggest the Roman province was in a desperate state of famine that confirms it’s relative weakness (Frend 1992). Thirdly, the seemingly coordinated nature of the ‘Conspiracy’ aligns more closely with an organised movement of strong onto weak, rather than a more chaotic assault had the invaders been in a state of desperation. We propose that the reconstructed drought conditions from 364 to 366 CE directly contributed to grain deficits in Roman Britain, which contributed to rebellions and subsequent military instability in the following year (Morris 2021). As a result of this military instability, tribes outside the periphery of the province were offered an opportune moment to invade the province in its weakened state. This mechanism is not without precedent, and numerous studies have linked the initiation of conflict and decline with drought and subsequent agricultural deficits (Esper et al. 2017; Gill et al. 2007; Pribyl et al. 2019).

Numerous questions remain around the scale and effect of the ‘Barbarian Conspiracy’, and why the drought from 364 to 366 CE have contributed to such a catastrophic event, particularly as the mid 4th century was less chaotic on the continent than the mid 3rd, or 5th, for instance. Part of the explanation lies in the unique positioning of Britain, physically isolated from the rest of the Empire, and thus experiencing external and internal pressure more acutely than the continental provinces (Higham 2004). The prolonged and extreme drought seems to have occurred during a particularly poor period for Roman Britain, in which food and military resources were being stripped for the Rhine frontier, while immigratory pressures increased. These factors limited resilience, and meant a drought induced, partial-military rebellion and subsequent external invasion were able to overwhelm the weakened defences. A database of coin hoards from late Roman Britain that we compiled from the British Museum (PAS 2023) affirms the likelihood that this was a highly chaotic period for the province (Fig. S1). Typically seen as a response to threat, coin data suggest that hoarding during the ‘Barbarian Conspiracy’ was not as severe as during other periods of conflict in late Roman Britain. The data, however, does coincide with a phase of somewhat elevated hoarding, which abruptly ended, possibly due to reestablishment of military control in 369 CE (Blockey 1980). See Fig. S1 and the attached text for a longer discussion of coin hoarding.

Further questions subsequently arise over why the Roman army, the primary mechanism of the Empire, was unable to effectively redistribute grain to meet deficits (as it had been to, for instance, the Rhine frontier), given its extensive trade network with even the most far-flung provinces (Dèry 1996). Yet precedent tells us that ‘war had the potential to disrupt even the most well-prepared supply routes’; in 48 BC Caesar's army ran out of wheat, while the armies of Septimius Severus experienced a similar catastrophe during the late 2nd century (Dèry 1996). Erdkamp (2019) indeed recognises that economic decline from the 3rd century in the Western Roman Empire meant its ability to alleviate harvest fluctuations through commercial and socio-political market mechanisms was increasingly limited. It is therefore certainly feasible that the isolation of Britain and the severity of the drought combined, as elsewhere in history, to cause such acute hardships, military breakdown, and the subsequent barbarian incursions (Slavin 2016).

### Climate and conflict across the Roman Empire

Our aggregate analysis suggests that dry and warm summers played a role on conflict inception in the Western Empire. The greatest number of years exceeding the dry threshold occurs three years prior to the ‘battle year’ (Fig. 3a and b), suggesting that dry and hot conditions had a lasting or cumulative impact on societal cohesion, and a lag existed as the effects took time to overwhelm societal and agricultural resilience. More recent studies have found different relationships; De Juan and Wegenast (2020) for instance, find that temperature and food riots are negatively correlated in 18th century Britain, implying inconsistent relationships over time and space. Interestingly, the OWDA-based record for the Western Empire (59 battles) shows no years crossing the drought-threshold during the ‘battle year’, which would suggest a return to normal, or even wetter than normal conditions, though this is not confirmed by the other climate records (Fig. 3a). This, in part, however, aligns with the finding of Tol and Wagner (2010) and Zhang et al. (2006) that cold and wet conditions increase the frequency of conflict in northern Europe and China, though the lag period between these conditions and conflict would be short (< 5 months).

Tol and Wagner (2010) also identify high spatial dependencies in climate-conflict interactions since 1000 CE; while cold and wet climates increased the frequency of conflict in northern Europe, in southern Europe hot and dry climates had the same effect (Ljungqvist et al. 2020). Similarly, we find contrasting results across the Empire; conflict in the East is preceded by a significant number of wetter summers, while temperature has no discernible relationship with the battle years. The Eastern Empire shows fewer statistically significant links to our climate records, and thus appears less reactive to extremes, which supports views of greater stability compared to the Western Empire as a result of an array of historical and political factors (Erdkamp 2019; Kaldellis 2024) (Fig. 3c, d). Across the entire late Roman Empire, we also see that conflict was heavily concentrated in spring and summer, which demonstrates the seasonality of conflict and the importance of mechanical considerations such as moving armies across wet ground (Büntgen and Di Cosmo 2016). This is a further link between climate and conflict, with repeated drier and hotter summers hardening the ground and allowing for easier movement of armies across terrain, as in modern conflicts (Wood 2011).

These investigations are hindered by the sparse paleo-climate proxy archives for the Eastern Roman Empire (Finné et al. 2011; Touchan et al. 2014). Additionally, as with all historical events, deteriorating political and economic factors in the Western Empire were undoubtedly strong contextual drivers for the ‘Barbarian Conspiracy’, and we recognise the danger in elevating the importance of one factor over a range of others (Erdkamp 2019). This study therefore makes clear not to attempt a simplification of the range and history of socio-political pressures that had been acting upon Roman Britain and the Western Roman Empire for centuries. Nevertheless, climate has repeatedly and valuably been demonstrated as a driver of societal change, and in that light we propose the 364–366 CE drought as an environmental catalyst that may explain the scale and abrupt nature of the event (Hsiang et al. 2013; Lee et al. 2015).

**Fig. 4:**
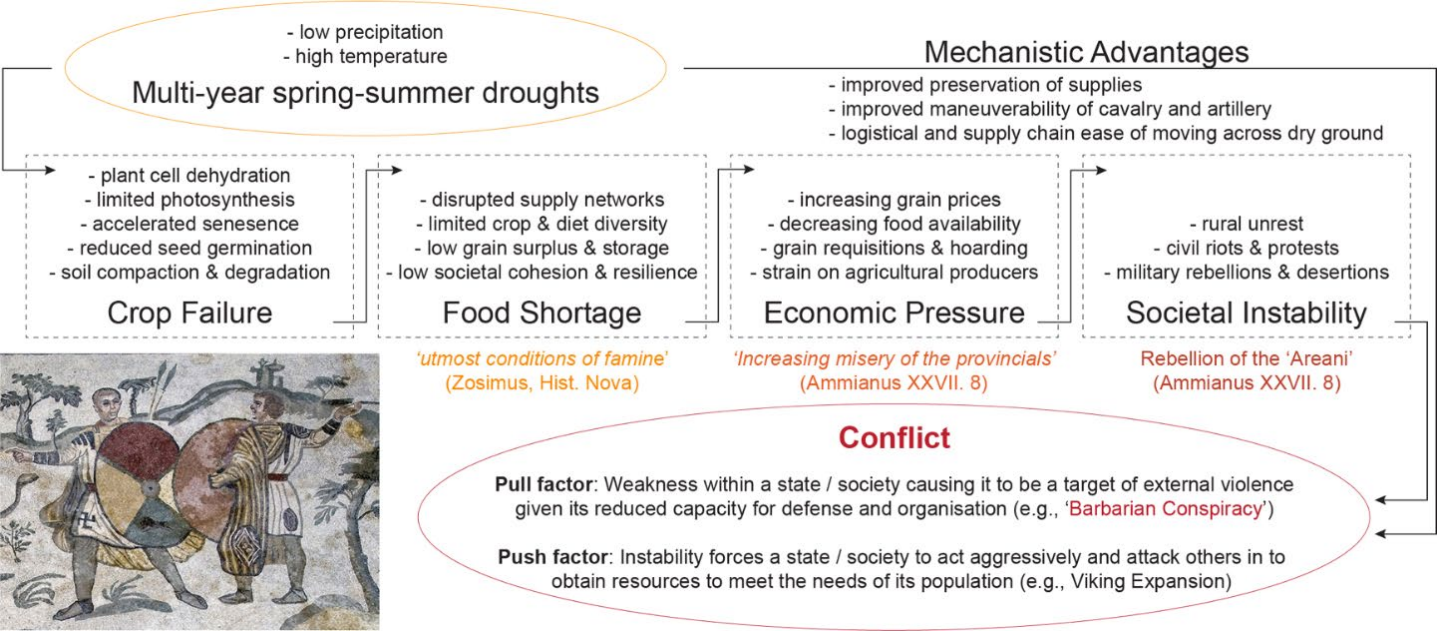
Schematic model for environment-conflict interactions based on the Barbarian Conspiracy of 367 CE and wider analysis of climate-conflict interactions in the late Roman period (350–476 CE). Model incorporates elements of climate, agriculture, ecology, environmental resilience, societal cohesion, mechanistic considerations and conflict initiation

Building on knowledge from the ‘Barbarian Conspiracy’ and the wider Roman Empire analysis, we create a climate-conflict causative model with agriculture as the main driver (Fig. 4). Similar and extensive literature exists on climate-conflict interactions in the present (Breckner and Sunde 2019), early modern (Degroot 2018) and medieval periods (Lee 2018). Here, we extend this discussion back to antiquity (Büntgen et al. 2016). The model attempts to recognise some potential environmental resilience, given that this is a critical factor in antiquity climate-conflict interactions (Erdkamp 2019; D’Angeli et al. 2022; Vivekananda et al. 2014). The components of the model are verified by literary sources on the ‘Barbarian Conspiracy’, such as the descriptions of famine and military rebellions by Ammianus (Ammianus XXVIII. 8.). Our mechanistic model proposes that a divergence of societal cohesion between central and peripheral societies resulting from food deficits causes conflict, either through ‘push’, or ‘pull’ factors, such as in the case of the ‘Barbarian Conspiracy’. Our model adds to the discussion on agriculture as a nexus between climate and conflict (Parker 2013; Warde 2015).

## Conclusion

This study provides an additional environmental dimension to the socio-political decline of Roman Britain and the wider Empire in the 4th and 5th centuries CE. This study further comes at a highly opportune moment; only recently have annually resolved and absolutely dated climate reconstructions and documentary sources from the late Roman Empire become available. Since a sequence of severe spring-summer droughts likely contributed to one of the most cataclysmic and damaging events in Roman Britain, the ‘Barbarian Conspiracy’ in 367 CE, the role of hydroclimate and environmental variation should receive more nuanced consideration in historical argumentation (Fig. 4). Our analysis of the entire Roman Empire further reveals exceptionally warm and dry summers in the Western Empire, and particularly wet summers in the Eastern Empire, often preceded military conflict. Increasingly detailed historical and paleo-environmental data are expected to further uncover complex entanglements between climate and society; in particular, new tree ring-based climate reconstructions covering the entire Common Era, along with synthesised documentary evidence from antiquity will increase the scope for further investigation.

## Electronic Supplementary Material

Below is the link to the electronic supplementary material.

Supplementary Material 1 (DOCX 0.99 MB)

## Data Availability

All data needed to evaluate the conclusions in the paper are present in the paper and/or the Supplementary Materials. The full battles record can be viewed at: https://www.academia.edu/101107871/Erhebungen_und_Empörungen_Machtkämpfe_und_Krisenbewältigung_im_spätrömischen_Westen. Further details of the data used in our precipitation reconstruction can be found in the Supplementary Materials of Cook et al. 2015. Requests and queries on data information should be submitted to Charles Norman, Capn2@cam.ac.uk.
