# Supplementary material for: Droughts and conflicts during the late Roman period
Source: Clim Change. 2025 Apr 16;178(5):87. doi: 10.1007/s10584-025-03925-4 (PMC12003598; doi:10.1007/s10584-025-03925-4)
Supplement: Supplementary file 1 — Supplementary Material 1 (DOCX 0.99 MB) [file 10584_2025_3925_MOESM1_ESM.docx]

Supplementary Materials for

# **Droughts and conflicts during the late Roman period**

**This file includes:**

Supporting text including coin hoarding and methodology discussion

Figures S1 to S3

Link to battles publication


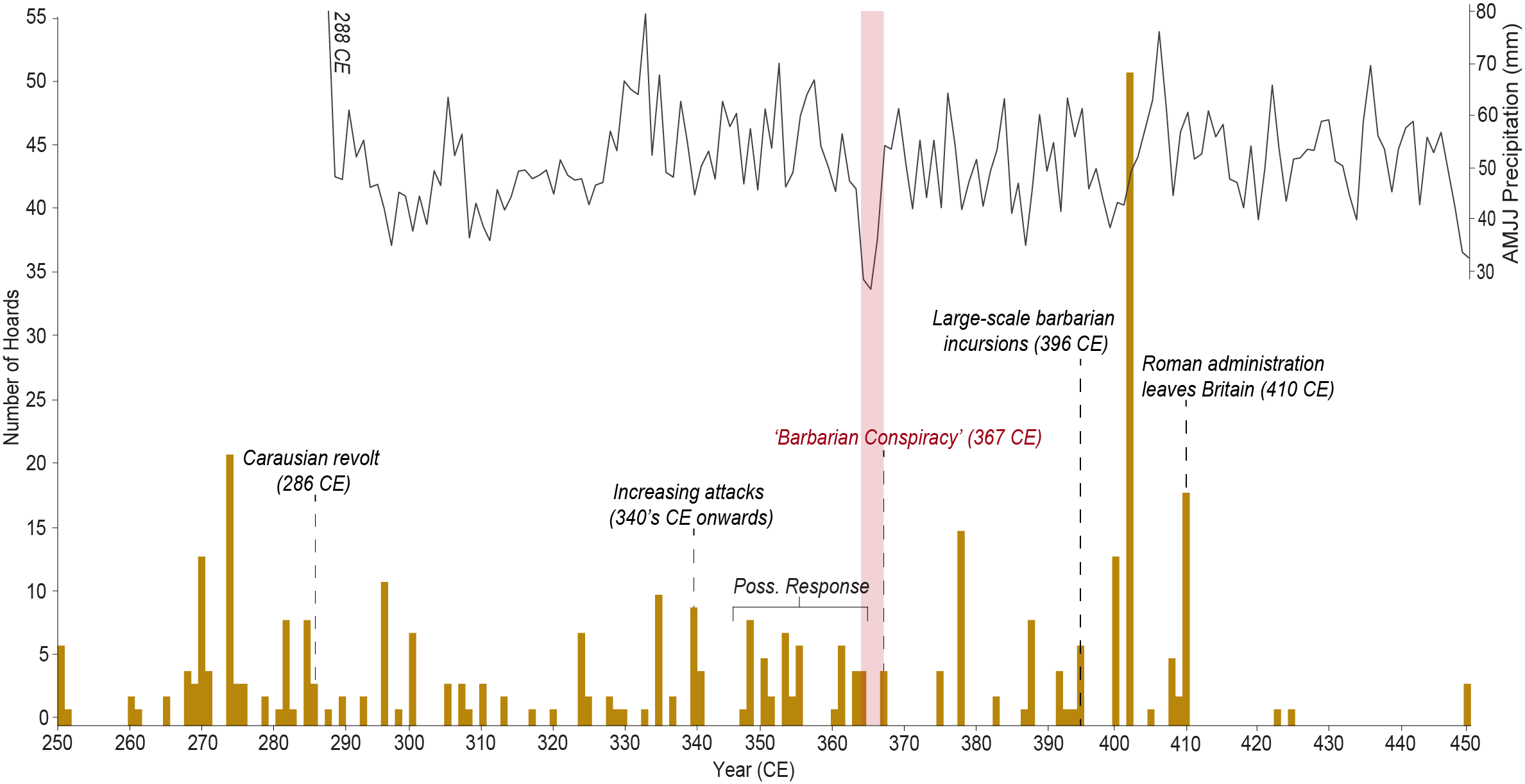


**Supplementary Figure 1.** Societal response to disorder in late Roman Britain as shown through total coin hoards per annum. Data from the Portable Antiquity Scheme of the British Museum (PAS, 2023). Year shown is the latest (most recent) datable coin from the hoard, indicating the last year in which it could have been buried. Most hoards are thought to contain coins from just a few years preceding the deposition, meaning here the potential response to an event such as the ‘Barbarian Conspiracy’ is shown before the event itself.

**Climate, conflict and coins**

Coin hoards are deposits of valuables, typically made in response to external threats with the intent of later recovery (Bland, 2018). Many in Roman Britain were not recovered, and those found can be used as a ‘barometer for unrest’ particularly amongst the wealthier classes (Morris, 2021). Hoards in Britain are not absolutely dated as the province lacked an imperial mint, and so most coinage came took a few years to enter circulation from the continent (Casey, 1984). The Hoxne Hoard for instance, the largest gold and silver hoard ever found in Britain, contains coins dated from, and is thus plotted to, 407 CE; it therefore had to be buried sometime after, though the lag period is unclear (Morris, 2021). The ‘response’ in hoarding to an event therefore appears prior to, rather than after the given date in **Figure S1**, which has the potential to lead to confusion in interpreting patterns. Our data nevertheless suggest that from 350–500 CE, coin hoarding responded dramatically to changes in societal conditions such as periods of conflict or destabilising political events (**Fig. S1**).

For instance, the greatest quantity of some 90 hoards falls in the 400s a decade which saw the Usurpation of Constantine III, troop withdraws to fight Alaric I, and the severance from Rome around 410 CE. Elsewhere, entanglements with climate can be proposed. The period from 294–304 CE was anonymously dry, according to this studies precipitation reconstruction and several others (**Fig. 1d**) (Büntgen et al., 2011; Cook et al., 2015), while historical sources note some form of Pictish uprising in northern Britain in the later years of this dry period, and in 305 CE Emperor Chlorus campaigned north of Hadrian’s wall (Barnes, 1981; Birley, 2005; Southern, 2004). Finally, the coin hoard record from 294–310 CE sees almost 30 hoards deposited, while the following same period sees less than half. The lack of detailed historical information makes establishing any clear relationships challenging, though this provides one instance where a prolonged dry period may have contributed to increased conflict around 300 CE and subsequent hoarding.

The ‘Barbarian Conspiracy’ does not see such a distinct jump in coin hoarding, though hoarding declines sharply afterwards likely as a result of re-established military control. Twenty-one hoards are deposited containing coins minted between 360–367 CE, compared to the following period of 368–374 with just four (PAS, 2023). The ‘Conspiracy’ thus appears to have resulted in less dramatic societal upheaval than other conflicts in Roman Britain, though it undoubtedly contributed to the high level of background hoarding during the 4^th^ century. This once again demonstrates the varying consequences of climate throughout time, strongly linked to the nature of subsequent conflict and resilience of the society. This furthers our contextual understanding of the ‘Barbarian Conspiracy’ and confirms the use of coin hoards as a possible proxy for investigating the climate-society-conflict nexus (Bland, 2018; Turchin and Scheidel, 2009).


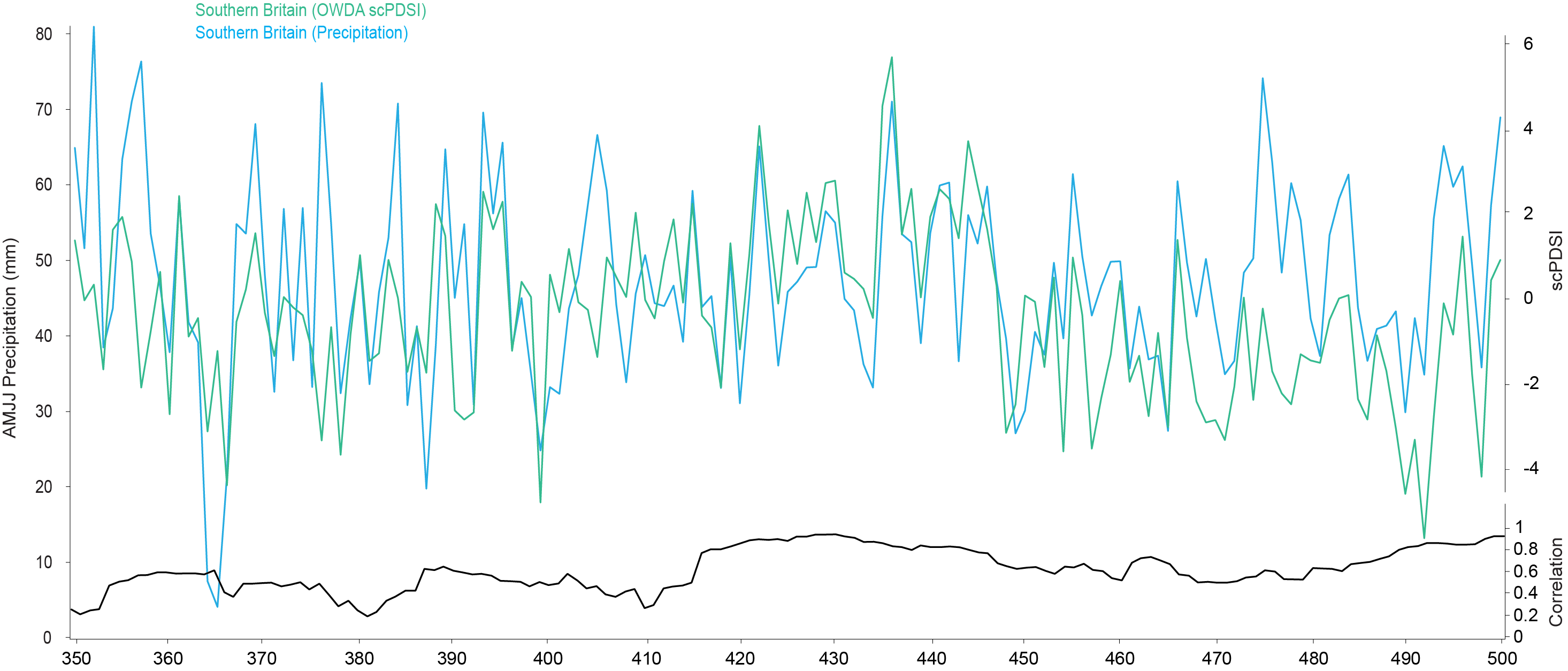


**Supplementary Figure 2**. Comparison of this studies precipitation reconstruction based of a *Quercus* TRW subset of the OWDA from southern Britain, and the OWDA scPDSI (drought) reconstruction of the same area. Black line shows moving correlation over a 21-year window. Correlation is low at the beginning of the timeseries in 350 CE at around 0.35, then rises relatively consistently until 500 CE at around 0.9. Correlation is also particularly high between 415 and 465 CE. The incorporation of temperature into the scPDSI reconstruction accounts for variation of the records and subsequent low correlation in certain places. The years 364, 365 and 366 CE are the most significant anomalies in the precipitation record, though are shown as less exceptional in the scPDSI record. This suggests that the dry period from 364–366 was largely precipitation driven, and temperatures were likely more typical.


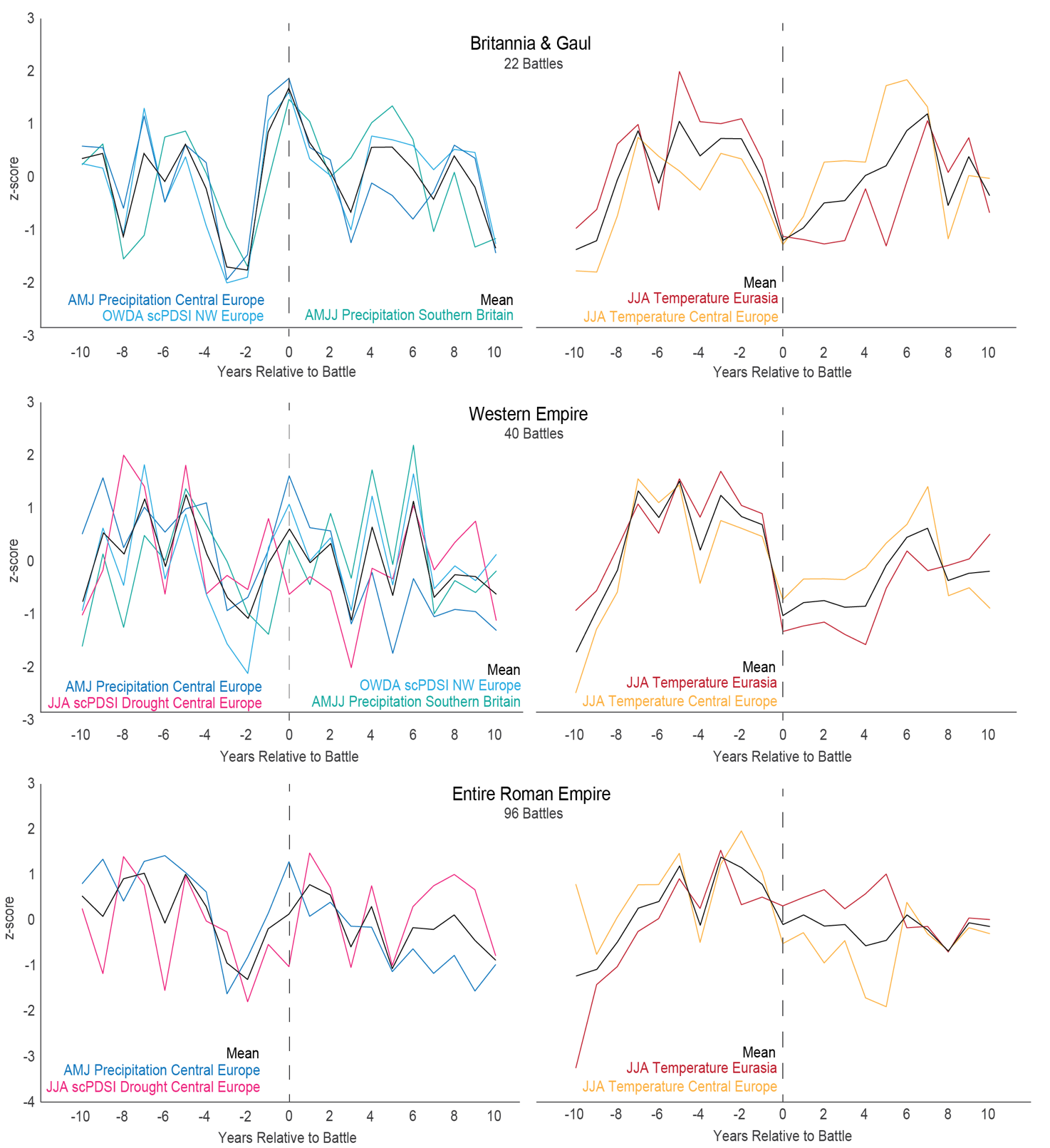


**Supplementary Figure 3.** A form of superposed epoch analysis used as a previous version of the climate-conflict analysis. Here, we compiled all battles for each region of interest; ‘Britannia and Gaul’ (northwest), Western Empire and Entire Roman Empire comprising 96 battles. For each region, we compiled the climate data for 10 years prior and after the battles for each conflict and climate reconstruction. These were overlain with each other, using the battle year as ‘year 0’, and the subsequent mean plotted as above. For instance, for Britannia and Gaul, the average climate of 22 battles suggested the common signal was a reduction in precipitation two to three years prior to the ‘battles year’. This iteration of analysis was replaced, as it couldn’t fully account for consecutive years with battles and failed to represent the climate signal preceding many battles.

**Battles Data**

Battles record including all known and dated large-scale battles in the late Roman Empire from 365–476 CE can be found here: <https://www.academia.edu/101107871/Erhebungen_und_Empörungen_Machtkämpfe_und_Krisenbewältigung_im_spätrömischen_Westen>

**References (supplementary material only)**

Barnes TD (1981) Constantine and Eusebius. Harvard University Press, Cambridge, Mass.

Birley AR (2005) The Roman government of Britain. Oxford University Press, Oxford.

Casey PJ (1984) Roman Coinage in Britain. Bloomsbury Shire Publications.

Turchin P, Scheidel W (2009) Coin hoards speak of population declines in ancient Rome. Proc Natl Acad Sci 106(41):17276-17279
